# Clinical Impact of Comprehensive Molecular Profiling in Adolescents and Young Adults with Sarcoma

**DOI:** 10.3390/jpm14020128

**Published:** 2024-01-23

**Authors:** Eden C. Andrew, Jeremy Lewin, Jayesh Desai, Lisa Orme, Anne Hamilton, Susie Bae, Wenying Zhu, Shannon Nicolson, Leila N. Varghese, Camilla B. Mitchell, Joseph H. A. Vissers, Huiling Xu, Sean M. Grimmond, Stephen B. Fox, Stephen J. Luen

**Affiliations:** 1 Department of Medical Oncology, Peter MacCallum Cancer Centre, Parkville, VIC 3000, Australia; 2 Children’s Cancer Centre, Royal Children’s Hospital, Parkville, VIC 3052, Australia; 3 Victorian Adolescent and Young Adult Cancer Service, Parkville, VIC 3000, Australia; 4 Sir Peter MacCallum Department of Oncology, The University of Melbourne, Melbourne, VIC 3010, Australia; 5 Department of Paediatrics, The University of Melbourne, Melbourne, VIC 3052, Australia; 6 Centre for Cancer Research and Department of Clinical Pathology, The University of Melbourne, Melbourne, VIC 3010, Australia; 7 Department of Pathology and Cancer Research Division, Peter MacCallum Cancer Centre, Parkville, VIC 3000, Australia; 8 Department of Clinical Pathology, The University of Melbourne, Melbourne, VIC 3010, Australia

**Keywords:** adolescents and young adults (AYAs) with cancer, molecular profiling, precision oncology, genomics, whole genome sequencing, next-generation sequencing, sarcoma, diagnostic biomarkers

## Abstract

Sarcomas are a heterogenous group of tumours that commonly carry poor prognosis with limited therapeutic options. Adolescents and young adults (AYAs) with sarcoma are a unique and understudied patient population that have only achieved modest survival gains compared to other groups. We present our institutional experience of AYAs with sarcoma who underwent comprehensive molecular profiling (CMP) via either large-panel targeted DNA sequencing or whole genome and transcriptome sequencing and evaluated the feasibility and clinical impact of this approach. Genomic variants detected were determined to be clinically relevant and actionable following evaluation by the Molecular Tumour Board. Clinicians provided feedback regarding the utility of testing three months after reporting. Twenty-five patients who were recruited for CMP are included in this analysis. The median time from consent to final molecular report was 45 days (interquartile range: 37–57). Potentially actionable variants were detected for 14 patients (56%), and new treatment recommendations were identified for 12 patients (48%). Pathogenic germline variants were identified in three patients (12%), and one patient had a change in diagnosis. The implementation of CMP for AYAs with sarcoma is clinically valuable, feasible, and should be increasingly integrated into routine clinical practice as technologies and turnaround times continue to improve.

## 1. Introduction

Comprehensive molecular profiling (CMP) via next-generation sequencing (NGS) is one of the cornerstones of precision oncology and can significantly impact clinical decision making. Sarcomas are a complex group of tumours with heterogenous biology that commonly carry poor prognoses with limited therapeutic options. The use of genomic profiling in sarcoma has the potential to improve patient outcomes through its capacity to improve our understanding of tumour biology, provide pathological evaluation for an accurate diagnosis, reveal targeted therapeutic options, and assist in accurate prognostication [1,2]. Historically, CMP was not considered a routinely feasible option given the barriers of access and expense; however, in the modern era of precision oncology, these technologies are rapidly becoming more accessible.

Adolescents and young adults (AYAs) with sarcoma have distinct biological and clinical features and, as a patient population, have been underrepresented in clinical trials [3]. The prognosis for AYA with sarcoma is typically inferior to their younger counterparts, and although underlying host and tumour factors likely contribute, this is poorly understood [4,5]. Moreover, the onset of cancers associated with genetic predisposition syndromes frequently occurs during AYA years. This underscores the imperative to invest in molecular profiling initiatives for this age group [6]. CMP is hypothesized to enhance clinical care for AYA patients with sarcoma through the identification of actionable genomic biomarkers and/or germline predisposition; however, clinical evidence to support its use is lacking [7].

The aim of this study was to describe the frequency of actionable variants and the feasibility and clinical impact of prospective CMP for AYA sarcoma patients at an adult tertiary referral sarcoma service.

## 2. Materials and Methods

This is an analysis of AYA patients with sarcoma whose tumour specimens underwent CMP via recruitment to the Victorian Comprehensive Cancer Centre (VCCC) PRECISION study. All included patients were treated and recruited at the Peter MacCallum Cancer Centre, Australia’s leading tertiary referral sarcoma service, between 1 July 2019 and 31 July 2023. The institution is a predominantly adult service, although patients as young as 15 years old are seen. Patients were eligible for inclusion in this study if they were aged between 15 and 39 years, which is consistent with major North American and European working group definitions of the AYA age range [3]. Other inclusion criteria included a histological diagnosis of sarcoma that was incurable but with a life expectancy of at least six months and an ECOG performance status of 0 or 1. The study was approved by the institutional human research ethics committee. All participating patients provided written informed consent.

Eligible participants underwent either targeted sequencing (TS) or whole genome and transcriptome sequencing (WGTS) of their tumour tissue. WGTS was performed according to our previously described methodology [8]. TS was performed using either the TruSight Oncology 500 Assay (Illumina) or an in-house-developed tumour–normal comprehensive targeted DNA panel test [9,10]. Both TS assays are designed to detect selected fusions. The choice of TS or WGTS was based on clinician discretion; however, WGTS was favoured as the test of choice if a newly obtained biopsy was possible. A newly obtained biopsy was performed where feasible; otherwise, archival tumour specimens were used (for TS only). A matched germline sample (peripheral blood) was additionally sequenced for all patients who underwent WGTS, as well as in a subset of patients who underwent TS.

Detected genomic variants were classified into tiers by the level of evidence based on clinical significance according to AMP/ASCO/CAP Guidelines [11]. Clinically relevant driver alterations were further assessed for actionability by the Molecular Tumour Board. The final report, including results of molecular analysis and potential clinical implications, was then issued to the participant’s treating clinician. The molecular reports were then reviewed in conjunction with the clinical data by investigators of this study to assess for clinical significance and the actionability of identified variants. “New potentially actionable variants” were defined as previously unidentified variants leading to a change in diagnosis and/or with therapeutic implications, meaning they could predict response or resistance to systemic therapy as per the OncoKB classification system (www.oncokb.org, accessed on 24 November 2023) of the levels of actionability [12]. Unless these criteria were met, other variants that contributed diagnostic information, such as genomic rearrangements, or other biological information, such as oncogenic drivers, were noted but not classified as “new potentially actionable variants”.

The prospective collection of clinical data was performed using questionnaires completed by treating clinicians at enrolment, including patient demographics, diagnostic and treatment information for sarcoma, known germline mutations, details of relevant previous molecular testing, ECOG performance status, and availability of an appropriate archival specimen. The treating clinician was invited to provide feedback regarding the utility and impact of molecular profiling three months after the report was issued. Clinicians were medical oncologists who may not have specific expertise in genetics. A retrospective review of the electronic medical record was conducted to extract additional clinical data, including details of diagnosis, previous treatments, and outcomes to further determine the clinical impact of CMP.

## 3. Results

### 3.1. Patient Characteristics

Between 1 July 2019 and 31 July 2023, 25 AYA patients with a histological diagnosis of sarcoma were recruited. Survival and disease status data were collected until 4 September 2023 and censored at death for a median follow up of 3.2 years (IQR 1.3–6.3). Thirteen males and twelve females were enrolled. The median age was 26.6 years (IQR 22.5–31.8) at diagnosis and 28.7 years (24.8–34.0) at enrolment for CMP. Twenty patients had soft tissue sarcomas (80%) with bone tumours comprising the remainder (20%). The majority of patients had received prior treatment with surgery (n = 20, 80%), radiation therapy (n = 14, 56%) and at least one line of systemic therapy (n = 15, 60%) prior to CMP (Table 1). Further details regarding treatments prior to consent for CMP are described in Supplementary Table S1.

At the time of sequencing, almost all AYA patients had metastatic disease (n = 24, 96%) and of these, 17 (68%) had primary progressive disease or were experiencing first recurrence. Three AYA patients were newly diagnosed at the time of recruitment to the study, two with a poor prognosis (cases 9 and 17) and one with two lesions of uncertain relationship (Case 24). The remaining five cases were recruited to the study at disease recurrence or later.

Five AYA patients had CMP performed previously (TS n = 2, RNA sequencing panel n = 2, circulating tumour DNA assay n = 1). Those patients previously tested via TS or ctDNA went on to undergo WGTS. Two AYA patients had known germline aberrations prior to sequencing (RB1 and TP53).

### 3.2. Details and Feasibility of Molecular Testing

**Figure 1:**
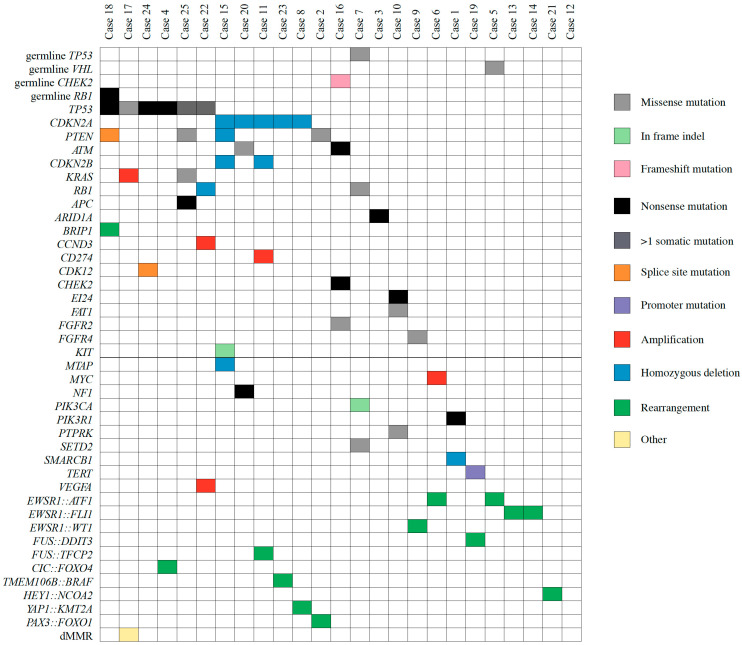
Driver variants identified via comprehensive molecular profiling. Legend—plot demonstrating driver variants and germline mutations identified across the study cohort.

Fifteen AYA patients underwent CMP via WGTS and 10 via TS. A new biopsy was required in half of the cases (n = 12, 48%). There were no patients who experienced a complication as a result of the biopsy. There were no cases of CMP assay failure. The median time from consent to the final molecular report was 45 days (IQR 37–57). Pathogenic variants predicted to drive tumour progression that were identified via CMP are described in Figure 1. As detailed in Table 2, more than half (n = 14, 56%) of the AYA patients who underwent CMP had a new potentially actionable variant identified. Among the 14 patients with new actionable variants, 12 had at least one new treatment recommendation and/or clinical trial option identified as a result of CMP, six of whom had more than one identified option. All treatment suggestions were based on Tier IIC or lower level of evidence.

^a^ High tumour mutational burden and microsatellite instability were considered actionable but were not included in the tiering system;
^b^ proposed treatment options were based on clinical trials available at the time of Molecular Tumour Board meeting;
^c^ identified based on whole transcriptome sequencing;
^d^ confirmed to be germline on subsequent testing.

Three patients commenced new treatments as a result of their molecular profiling, two of whom underwent treatment via enrolment in a clinical trial (Case 9: TRK inhibitor for 2.7 months and then ceased due to progressive disease; Case 18: combination PARP inhibitor and anti-PD1 antibody for 2.6 months and then ceased due to progressive disease). The third case (Case 23) had a favourable initial response to treatment with cobimetinib (by compassionate access) after a *BRAF* rearrangement was identified in a primary pancreatic spindle cell sarcoma; however, this case had progressive disease at 3.8 months and died approximately 5 months after treatment commenced [13]. The remaining nine AYAs who had new treatments identified did not commence treatment for the following reasons: clinical circumstances precluded their eligibility to enrol in a clinical trial (n = 2); death due to an unexpected severe medical complication (n = 2); unable to access recommended treatment (n = 1); treating clinician’s discretion (n = 1); patient preference (n = 1); and systemic therapy was not indicated because the patient was able to achieve remission via standard therapies (n = 2) (Supplementary Table S2).

Two AYA patients had germline variants detected through molecular profiling. Case 5 had a germline *VHL* missense variant of uncertain significance identified and Case 16 had a pathogenic germline *CHEK2* frameshift deletion. Both patients remained in complete remission at the time of follow up. Case 24 had a *TP53* deletion detected through molecular profiling via TS, which in combination with a strong family history led to subsequent germline testing that was confirmatory for Li–Fraumeni syndrome.

One patient had their histological diagnosis revised as a consequence of molecular profiling. On a background of multiply recurrent desmoid fibromatosis and newly progressive metastatic disease, a further biopsy was performed, which was sent for both an RNA sequencing fusion panel as well as WGTS. Both tests identified a *YAP1::KMT2A* fusion, which was supportive of a diagnosis of a rare subtype of sclerosing epithelioid fibrosarcoma (MUC4-negative during immunohistochemistry) [14]. WGTS also led to the refinement of diagnosis through the identification of gene fusions and/or fusion partners following initial testing with FISH break-apart probes in eight cases (32%). The details of gene fusion and fusion partners are shown in Figure 1.

### 3.3. Clinician Feedback

Three months after the molecular report was issued, the treating clinicians provided feedback regarding their perception of the clinical impact of the CMP testing results. Clinical feedback analysis was provided and evaluated for 22 patients. In two-thirds of these cases (n = 14, 64%), the treating clinician found that molecular profiling provided useful information aside from deciding the current therapy. The most frequent reasons for this included the following: the identification of future treatment options (n = 8), clarifying the diagnosis (n = 7), avoiding treatments (n = 3), and clarifying or refining prognosis (n = 2). Of the 11 cases where a potentially actionable variant was not identified, clinician feedback was available for 10 cases, 3 of whom found testing useful despite not finding an actionable variant.

## 4. Discussion

In this series, we demonstrate the clinical utility of CMP among a cohort of AYA patients with sarcoma, most of whom had advanced and poor prognosis disease. At least one newly identified potentially actionable variant was identified in more than half of the cohort (n = 14, 56%), with the majority of these discoveries translating to new therapeutic options (n = 12, 48%).

Our results are consistent with other reports; however, the frequencies of the identification of variants with therapeutic potential vary widely. To our knowledge, there is only one other published study focusing on CMP exclusively for AYA sarcoma patients. In their multisite European cohort of 48 patients, a very high frequency of actionable variants with therapeutic recommendations (81%) was identified by a combination of whole exome sequencing, methylation profiling, and RNA sequencing [7]. More consistent with our study, studies on older adults using a variety of NGS platforms reported finding biomarkers with therapeutic potential in 36–56% of participants [1,15,16,17]. A large study of 7494 sarcoma patients of all ages reported a lower overall rate of 31% and did not identify specific differences for the younger cohort (≤30 years), although AYA patients were not separated as a subgroup [18]. The variation in results is likely multifactorial and reflective of the heterogenous patient and tumour group, differences in testing platforms and assays, selection bias, and the challenges of the interpretation and classification of actionable variants that lend themselves to guiding therapeutic choices [19]. Within this study, of those patients who were tested using WGTS, 11/15 (73%) had actionable mutations, compared with only 3/10 (30%) of those tested using TS. Whilst this observation is noteworthy, it remains unclear which AYA subgroups should preferentially use WGTS. It should be noted that most TS panel tests do not include the ability to detect rarer fusions which are enriched in AYA sarcomas.

The implementation of CMP in our cohort was highly feasible. Biopsies were repeated when required, and testing occurred with universal success and without complication. The median turnaround time of six weeks from enrolment to reporting is shorter than other reports [7], although in the clinical context of poor prognosis disease with limited options for effective treatments, six weeks is significant and argues for implementing CMP earlier in the clinical journey. Of the 12 patients with new treatment options identified via CMP, only 3 were able to start this therapy. This is likely linked to the turnaround time and barriers to drug accessibility and because testing occurred relatively late in the patient’s journey [20].

The majority of patients included in this study underwent testing when standard treatments were no longer effective, with only three patients having CMP at diagnosis. Performing upfront CMP at the diagnosis of a metastatic or poor prognostic sarcoma, especially when the planned standard of care treatment does not depend on the results, would allow for the advanced planning of personalised second-line and salvage therapies. However, whilst our experience advocates for earlier CMP implementation, consensus is lacking regarding the best time to perform molecular profiling. A relative lack of targeted therapeutic options at least in part contributes to this, as well as the understanding that changes in tumour biology such as clonal diversity and various mechanisms of treatment resistance occur over time [21,22]. Other factors contributing to a reluctance to test earlier may include clinical concerns around the risks of repeated biopsy, the cost of testing and lack of consensus around which patients would benefit most, as well as which testing platforms to utilise.

We add to a body of evidence demonstrating the clinical value of CMP beyond the ability to identify new treatment options [23,24]. Of major importance to AYA patients, CMP led to the identification of pathogenic germline variants in 12% of our cohort, which is similar to previous reports in this population [25,26], although this is notably less than the finding of 55% in a large multisite cohort of 1162 adult sarcoma patients [27]. Our department now advocates for referral to a familial cancer centre to offer germline testing for all patients under 40 years with sarcoma, as well as for older patients with a suggestive personal or family history. Whilst only one patient had a formal change in diagnosis, CMP allowed diagnoses to be refined in several cases which was still deemed useful for clinicians. For example, the identification of the exact oncogenic fusion driver in a tumour explained specific patterns of treatment resistance and poor prognosis.

A strength of our study is the inclusion of clinician perspectives regarding the clinical value of CMP for their AYA patients. The majority of clinicians found CMP to be useful aside from making decisions about current treatment. The fact that several clinicians found profiling to be useful even following negative tests due to the perceived benefits of clarifying diagnosis and avoiding future treatments underscores the clinical value of CMP as a tool to improve care for AYA sarcoma patients.

Despite their unique biological and clinical care needs, AYA patients tend to fall in a gap between traditional adult and paediatric approaches. In the adult oncology setting where there is a much higher volume of patients, the use of CMP tends to be more judicious and typically reserved for those with advanced disease and with a high pre-test probability of identifying a molecular target. In contrast, in the paediatric setting in Australia and internationally, precision oncology efforts are now moving towards offering CMP to all children with a new cancer diagnosis, irrespective of disease extent or prognosis, and they are expanding programs to include platforms such as in vitro drug testing, patient-derived xenograft models and phosphoproteomics in order to improve personalised treatment recommendations [28,29]. These differences in approaches are reflective of known differences in biology between paediatric and adult sarcomas; however, sarcoma subtypes experienced by AYA patients can span this divide and would greatly benefit from an individualized approach [30].

This study is limited by its small sample size, single-site design, and potential selection bias from treating clinicians. Another important limitation of this and other studies in this field is the absence of patient-reported outcomes, which is especially important in AYA research [31]. The patient’s perspective of the experience of the process and outcomes of CMP could considerably contribute to assessments of clinical value and utility. High levels of satisfaction and perceived benefit have been reported by parents and adolescent patients enrolled in precision oncology programs in the paediatric setting [32]. Further, in view of the different definitions of AYA used internationally, it should be noted that the median age of 26.6 years in this study potentially corresponds to a different spectrum of diagnoses and associated genomic findings compared with other younger cohorts, such as those restricted to less than 25 years old.

There is an urgent need to enable access to clinical trials for AYA patients, especially those with a molecular focus, in order to improve overall care and outcomes for this vulnerable group. The results of current adult and paediatric clinical trials are eagerly awaited to inform whether CMP in sarcomas can identify new promising therapeutic targets, whether this can be integrated into clinical practice, and whether this translates to improved patient outcomes, although dedicated research specifically for the AYA group is needed [29,33]. Nevertheless, we identified that the implementation of CMP for AYA with sarcoma is clinically valuable and feasible and should be increasingly integrated into routine clinical practice as technologies, cost, and turnaround times continue to improve.

## Figures and Tables

**Table 1:** Patient and tumour characteristics.

Case	Age (y) at Diagnosis	Sex	Histological Diagnosis	Primary Tumour Site	Disease Extent at Diagnosis	Lines of Systemic Treatment	Other Prior Treatment	Sequencing Platform
1	22.9	M	Alveolar rhabdomyosarcoma	Head and neck	Localised	2	Surgery, RT	WGTS
2	26.6	M	Alveolar rhabdomyosarcoma	Head and neck	Metastatic	1	RT	TS
3	34.7	F	Angiosarcoma	Thorax	Localised	1	Surgery, RT	TS
4	27.5	M	CIC-rearranged sarcoma	Extremity	Metastatic	1	RT	WGTS
5	22.6	M	Clear cell sarcoma of soft tissue	Abdomen	Localised	0	Surgery	WGTS
6	20.3	M	Clear cell sarcoma of soft tissue	Extremity	Localised	0	Surgery, RT	WGTS
7	29.4	M	Dedifferentiated chondrosarcoma	Pelvis	Localised	1	Surgery	TS
8	19.6	F	Desmoid fibromatosis, recurrent	Extremity	Localised	2	Surgery, RT	WGTS
9	21.5	M	Desmoplastic small round cell tumour	Abdomen	Metastatic	1		WGTS
10	23.0	F	EBV-associated smooth muscle tumour	Abdomen	Metastatic	2	Surgery, RT	WGTS
11	34.4	F	Epithelioid sarcoma	Extremity	Localised	2	Surgery, RT	TS
12	31.3	F	Epithelioid sarcoma	Pelvis	Metastatic	0	Surgery, RT	TS
13	15.7	F	Ewing sarcoma	Thorax	Localised	2	Surgery, RT	TS
14	31.8	M	Ewing sarcoma	Head and neck	Metastatic	4	Surgery, RT	TS
15	24.4	F	Gastrointestinal Stromal Tumour	Abdomen	Metastatic	3	Surgery	WGTS
16	21.7	M	Hepatic sarcoma, NOS	Abdomen	Localised	0	Surgery	WGTS
17	31.8	F	Intimal Sarcoma of Pulmonary Artery	Thorax	Localised	0	Surgery	WGTS
18	23.7	M	Leiomyosarcoma—radiation induced	Head and neck	Localised	0	Surgery	WGTS
19	34.7	M	Malignant peripheral nerve sheath tumour	Head and neck	Localised	0	Surgery, RT	TS
20	27.6	F	Mesenchymal chondrosarcoma	Pelvis	Metastatic	1	RT	WGTS
21	32.5	F	Myxoid liposarcoma	Extremity	Localised	0	Surgery, RT	WGTS
22	20.9	F	Osteosarcoma	Extremity	Metastatic	1	Surgery	WGTS
23	22.5	F	Primary pancreatic sarcoma, NOS	Abdomen	Localised	0	Surgery	WGTS
24	33.9	M	Spindle cell sarcoma, NOS	Thorax	Metastatic	0	Surgery	TS
25	28.2	M	Teratoma with sarcomatous transformation, NOS	Thorax	Localised	1	Surgery	TS

M—male; F—female; NOS—not otherwise specified; RT—radiation therapy; WGTS—whole genome and transcriptome sequencing; TS—targeted sequencing.

**Table 2:** Newly identified potentially actionable variants and treatment recommendations.

Case	Histological Diagnosis	Sequencing Platform	New Potentially Actionable Variant(s)	AMP/ASCO Tiers for Clinical Significance ^a^	Proposed Treatment Options ^b^	Treatment Started
1	Alveolar rhabdomyosarcoma	WGTS	*CD274* amplification *CDKN2A* homozygous deletion	III III	Anti PD-1/PD-L1 antibody	No
5	Clear cell sarcoma of soft tissue	WGTS	Germline *VHL* missense variant	N/A		
6	Clear cell sarcoma of soft tissue	WGTS	*MYC* amplification	IIC	RNA Polymerase I inhibitor	No
7	Dedifferentiated chondrosarcoma	TS	*PIK3CA* in-frame deletion	IIC	PI3-kinase inhibitor	No
8	Desmoid fibromatosis, recurrent	WGTS	*YAP1::KMT2A* fusion *CDKN2A* homozygous deletion SBS Mutation Signature 3	IIC IIC N/A	TEAD inhibitor CDK4/6 inhibitor + checkpoint blockade PARP inhibitor + checkpoint blockade	No
9	Desmoplastic small round cell tumour	WGTS	*FGFR4* missense variant High *NTRK3* expression ^c^	IIC N/A	Pan TRK inhibitor	Yes
15	Gastrointestinal Stromal Tumour	WGTS	*CDKN2A*	IIC III	CDK4/6 inhibitor + checkpoint blockade	No
16	Hepatic sarcoma, NOS	WGTS	Germline *CHEK2* frameshift deletion *ATM* inactivating variant *FGFR2* missense variant	N/A III III	PARP inhibitor + checkpoint blockade FGFR inhibitor	No
17	Intimal Sarcoma of Pulmonary Artery	WGTS	High tumour mutational burden High microsatellite instability	N/A N/A	Anti PD-1/PD-L1 antibody	No
18	Leiomyosarcoma—radiation induced	WGTS	*BRIP1* rearrangement	IIC	PARP inhibitor + checkpoint blockade	Yes
19	Malignant peripheral nerve sheath tumour	TS	*NF1* deletion *ATM* substitution *CDKN2A* deletion	IIC IIC IID	MEK + mTOR inhibition PARP inhibitor + checkpoint blockade CDK4/6 inhibitor + checkpoint blockade	No
21	Myxoid liposarcoma	WGTS	*TERT* promoter variant	IID	None	
23	Primary pancreatic sarcoma, NOS	WGTS	*TMEM106B::BRAF* fusion	III	RAF dimer inhibitor + MEK inhibitor	Yes
24	Spindle cell sarcoma, NOS	TS	*TP53* deletion ^d^ *CDK12* substitution	IIC IID	CDK4/6 inhibitor + checkpoint blockade	No

WGTS—whole genome and transcriptome sequencing; PD-L1—programmed death-ligand 1; TS—targeted DNA sequencing; NOS—not otherwise specified; N/A—not applicable.

## Data Availability

The data presented in this study are available upon request from the corresponding author and after approval from the VCCC PRECISION study investigators. The data are not publicly available due to ethics limitations.

## Supplementary Materials

The following supporting information can be downloaded at: https://www.mdpi.com/article/10.3390/jpm14020128/s1, Table S1: Details of disease-directed treatment prior to consent for comprehensive molecular profiling; Table S2: Patients with an identified treatment target and reasons for not commencing proposed treatment.
