# Supplementary material for: Clinical Impact of Comprehensive Molecular Profiling in Adolescents and Young Adults with Sarcoma
Source: J Pers Med. 2024 Jan 23;14(2):128. doi: 10.3390/jpm14020128 (PMC10890624; doi:10.3390/jpm14020128)
Supplement: Supplementary file 1 [file jpm-14-00128-s001.zip › jpm-2774353-supplementary.pdf]

Supplementary Table S1 – Details of disease-directed treatment prior to consent for comprehensive molecular profiling

Case	*Age (y) at Diagnosis*	*Sex*	*Histological Diagnosis*	*Primary Tumour Site*	*Disease Extent at Diagnosis*	*Lines of Systemic Treatment*	*Prior Treatment*
1	22.9	M	Alveolar rhabdomyosarcoma	Head & neck	Localised	1	1st line: VAC chemotherapy, RT and surgery<br>2nd line: VIT chemotherapy, RT
2	26.6	M	Alveolar rhabdomyosarcoma	Head & neck	Metastatic	1	1st line: IVADo chemotherapy and RT
3	34.7	F	Angiosarcoma	Thorax	Localised	1	1st line: Surgery and RT<br>2nd line: Paclitaxel chemotherapy
4	27.5	M	CIC-rearranged sarcoma	Extremity	Metastatic	1	1st line: VDC/IE chemotherapy and RT<br>2nd line: Surgery
5	22.6	M	Clear cell sarcoma of soft tissue	Abdomen	Localised	0	1st line: Surgery
6	20.3	M	Clear cell sarcoma of soft tissue	Extremity	Localised	0	1st line: Surgery and RT
7	29.4	M	Dedifferentiated chondrosarcoma	Pelvis	Localised	1	1st line: Ifosfamide/doxorubicin chemotherapy and surgery
8	19.6	F	Desmoid fibromatosis, recurrent	Extremity	Localised	1	1st line: Surgery<br>2nd line: RT, liposomal doxorubicin chemotherapy
9	21.5	M	Desmoplastic small round cell tumour	Abdomen	Metastatic	1	1st line: VDC/IE chemotherapy
10	23.0	F	EBV-associated smooth muscle tumour	Abdomen	Metastatic	1	1st line: Everolimus and RT<br>2nd line: EBV-CTL and RT<br>3rd line: Surgery
11	34.4	F	Epithelioid sarcoma	Extremity	Localised	2	1st line: Surgery and RT<br>2nd line: Imatinib and surgery<br>3rd line: Surgery, RT, gemcitabine/docetaxel
12	31.3	F	Epithelioid sarcoma	Pelvis	Metastatic	0	1st line: Surgery and RT<br>2nd line: Surgery
13	15.7	F	Ewing sarcoma	Thorax	Localised	2	1st line: VIDE then VAI chemotherapy, surgery and RT<br>2nd line: VTC chemotherapy, autologous stem cell transplant (treosulfan/ melphalan) and RT<br>3rd line: RT
14	31.8	M	Ewing sarcoma	Head & neck	Metastatic	3	1st line: Surgery, RT and VDC/IE chemotherapy<br>2nd line: Surgery, VTC chemotherapy<br>3rd line: Surgery, RT, autologous stem cell transplant (busulfan/melphalan)<br>4th line: Oral etoposide
15	24.4	F	Gastrointestinal Stromal Tumour	Abdomen	Metastatic	1	1st line: Surgery and imatinib<br>2nd line: Sunitinib<br>3rd line: Ripretinib

16	21.7	M	Hepatic sarcoma, NOS	Abdomen	Localised	0	1st line: Surgery
17	31.8	F	Intimal Sarcoma of Pulmonary Artery	Thorax	Localised	0	1st line: Surgery
18	23.7	M	Leiomyosarcoma - radiation induced	Head & neck	Localised	0	1st line: Surgery
19	34.7	M	Malignant peripheral nerve sheath tumour	Head & neck	Localised	0	1st line: Surgery and RT
20	27.6	F	Mesenchymal chondrosarcoma	Pelvis	Metastatic	1	1st line: VDC/IE chemotherapy and RT
21	32.5	F	Myxoid liposarcoma	Extremity	Localised	0	1st line: Surgery and RT<br>2nd line: Surgery
22	20.9	F	Osteosarcoma	Extremity	Metastatic	1	1st line: MAP chemotherapy and surgery<br>2nd line: Surgery
23	22.5	F	Primary pancreatic sarcoma, NOS	Abdomen	Localised	0	1st line: Surgery
24	33.9	M	Spindle cell sarcoma, NOS	Thorax	Metastatic	0	1st line: Surgery
25	28.2	M	Teratoma with sarcomatous transformation, NOS	Thorax	Localised	1	1st line: Surgery

RT – Radiation Therapy VAC – Vincristine, Dactinomycin, Cyclophosphamide VIT – Vincristine, Irinotecan, Temozolomide
IVADo – Ifosfamide, Vincristine, Dactinomycin, Doxorubicin VDC/IE – Vincristine, Doxorubicin, Cyclophosphamide, Ifosfamide, Etoposide
EBV-CTL – Epstein-Barr Virus-Specific Cytotoxic T Lymphocyte infusions VIDE – Vincristine, Ifosfamide, Doxorubicin, Etoposide
VAI - Vincristine, Dactinomycin, Ifosfamide VTC - Vincristine, Topotecan and Cyclophosphamide MAP – Methotrexate, doxorubicin and cisplatin

Supplementary Table S2 – Patients with an identified treatment target and reasons for not commencing proposed treatment

Case	*Histological Diagnosis*	*Sequencing Platform*	*New Potentially Actionable Variant(s)*	*Proposed Treatment Options*	*Reason Targeted Therapy Not Commenced*
1	Alveolar rhabdomyosarcoma	WGTS	*CD274* amplification<br>*CDKN2A* homozygous deletion	Anti PD-1/PD-L1 antibody	Patient preference
6	Clear cell sarcoma of soft tissue	WGTS	*MYC* amplification	RNA Polymerase I inhibitor	Clinician preference
7	Dedifferentiated chondrosarcoma	TS	*PIK3CA* in-frame deletion	PI3-kinase inhibitor	Clinical circumstances precluded eligibility to enroll on a clinical trial

8	Desmoid fibromatosis, recurrent	WGTS	*YAP1::KMT2A* fusion<br>*CDKN2A* homozygous deletion<br>SBS Mutation Signature 3	TEAD inhibitor<br>CDK4/6 inhibitor + checkpoint blockade<br>PARP inhibitor + checkpoint blockade	Clinical circumstances precluded eligibility to enroll on a clinical trial
15	Gastrointestinal Stromal Tumour	WGTS	*CDKN2A*	CDK4/6 inhibitor + checkpoint blockade	Unable to access recommended treatment
16	Hepatic sarcoma, NOS	WGTS	Germline *CHEK2* frameshift deletion<br>*ATM* inactivating variant<br>*FGFR2* missense variant	PARP inhibitor + checkpoint blockade<br>FGFR inhibitor	Complete remission achieved with standard therapies
17	Intimal Sarcoma of Pulmonary Artery	WGTS	High tumour mutational burden<br>High microsatellite instability	Anti PD-1/PD-L1 antibody	Death due to unexpected severe medical complication
19	Malignant peripheral nerve sheath tumour	TS	*NF1* deletion<br>*ATM* substitution<br>*CDKN2A* deletion	MEK + mTOR inhibition<br>PARP inhibitor + checkpoint blockade<br>CDK4/6 inhibitor + checkpoint blockade	Death due to unexpected severe medical complication
24	Spindle cell sarcoma, NOS	TS	*TP53* deletion<br>*CDK12* substitution	 CDK4/6 inhibitor + checkpoint blockade	Complete remission achieved with standard therapies
